# Molecular docking analysis of cellulose from Bacillus species with cellotetraose

**DOI:** 10.6026/973206300210947

**Published:** 2025-05-31

**Authors:** Nikita Chordia Golchha, Pratibha Maravi, Hasanain Abdulhameed Odhar, Afreen Shaikh

**Affiliations:** 1 Department of Computer Science and Biosciences, Shri Vaishnav Institute of Management and Science, Gumasta Nagar, Indore- 452009, India; 2 Department of Botany, Government Holkar Science College, Bhawarkuan, A.B. Road, Indore-452001, India; 3 Department of Pharmacy, Al-Zahrawi University College, Karbala, Iraq; 4 Department of Biosciences, Mata Gujri College of Professional Studies, A.B. Road, Indore- 452001, India

**Keywords:** Cellulase, cellobiose, cellotriose, Cellotetraose, laminaribiose, docking, binding, endocellulase

## Abstract

Cellulose is an unbranched glucose polymer and has great potential for bioconversion to value-added bio products. Cellulose is the most dominating agricultural waste that can be degraded by cellulase which is produced by cellulolytic bacteria such as the Bacillus species. The binding efficacy with the substrate suggests the choice of the substrate that can be used for various industrial applications such as biofuel production, food and feed industry, brewing, pulp and paper, textile, laundry and agriculture. Therefore, it is of interest to describe the molecular docing analysis of cellobiose, cellotetriose, cellotetraose and laminaribiose with different cellulosederivatives such as endo-1, 4-beta-D-glucanase, exo-1, 4-beta-D-glucanase and beta-glucosidase. Thus, the molecular binding features of cellulase with cellotetraose are reported.

## Background:

Cellulose is the renewable biological resource and mostample biopolymer in the world. As non-renewable resources are becoming scarce, cellulose can be used to meet future needs for food, energy, fuel and other products [1]. Cellulose is degraded into glucose by breaking β-1, 4-glycosidic bond using an enzyme cellulase produced with the aid of both bacteria and fungi. Microorganisms produce mainly three types of cellulosederivatives namely-endo-1, 4-beta-D-glucanase, exo-1, 4-beta-D-glucanase and beta-glucosidase--either separately or in the form of a complex [2]. Naturally, cellulose is hydrolysedusing synergestic action of several enzymes whichinvolves its degradation from crystalline and/or amorphous cellulose to individual cellulose polysaccharides using endoglucanse. The exoglucanse or cellobiohydrolases act on the reducing and non-reducing ends of the exposed polysaccharide chains resulting in predominantly disaccharides (cellobiose) and lesser extent trisaccharides or tertrasaccharides. This are further hydrolyze by beta-glucosidase or cellobiases to glucose [3]. Cellulase are used in lots of industrial applications such as biofuel production, food and feed industry, brewing, pulp and paper, textile, laundry and agriculture [4]. Despite the proven role of cellulase for the industry, the inner mechanisms of cellulose degradation and its regulation are not fully characterized. Many bacteria have been widely explored for cellulose production among them, Bacillus species. Continues to be dominant due to the capacity to produce and secrete large quantities of extracellular enzymes [5]. The use of bacteria for such bio cellulosicconversion can leads to the "greener technology" [6]. In this study, we have docked all three components of cellulase with the different polysaccharides sub units namely, cellobiose, cellotetraose, cellotriose and laminaribiose. This will help in understanding the binding efficiency of different cellulase enzyme with the polysaccharides [7]. The results will also help in knowing different cellulase behaviour with different polysaccharides. It is important for future studies including efforts to bioengineer more efficient cellulase enzyme systems for industrial applications [8]. Therefore, it is of interest to describe the molecular docing analysis of cellobiose, cellotetriose, cellotetraose and laminaribiose with different cellulosederivatives such as endo-1, 4-beta-D-glucanase, exo-1, 4-beta-D-glucanase and beta-glucosidase.

## Methodology:

The three dimensional structure of endocellulase, exocellulase and beta-glucosidase of Bacillus species were downloaded from Protein databank [9]. Similarly, structure of polysaccharides sub units namely, cellobiose, cellotetraose, cellotriose and laminaribiose were downloaded from Pubchem database [10]. The downloaded structure were visualized and converted into docking file format using PyMol [11]. Docking studies were performed for each cellulase with different polysaccharides subunits using Autodock 4.2.6 [12]. AutoDockTools (ADT) software was used to prepare the input files [13]. First the cellulase macromolecule was prepared by deleting all crystallographic water molecules, metal ions and associated ligands. Thereafter, polar hydrogens and Gasteiger charges were added to macromolecule. Polysaccharide subunits as ligand was then prepared by detection of torsion tree root. When both macromolecule and ligand was prepared, grid file was prepared with default grid spacing of 0.375 Å and taking grid on the whole cellulase (blind docking). Docking parameter file was prepared in which, the macromolecule was kept rigid while the ligand was allowed to sample the specified torsional parameters. The docking calculations used were the standard auto dock force field and the Lamarckian geneticalgorithm to searchfor the best docked ligand conformers. Each docking experiment consisted of 50 independent runs with a population size of 150 and a random initial geometry for the ligand. The maximum number of energy evaluations for each run was set at 25,000,000 with the maximum number of generations ranging from 1000 to 27000 depending on convergence. The maximum number of top individuals that automatically survived was set to 1, the mutation rate was set to 0.02, the crossover rate was set to 0.8 and translational step size was set to 2 Å. Using the above parameter, docking was done for all three cellulase viz., endocellulase, exocellulase and beta-glucosidase with all four polysaccharides namely cellobiose, cellotetraose, cellotriose and laminaribiose. The results were noted for all twelve docking and compared for binding energy, ligand efficiency, total energy and hydrogen bonds formed.

## Results and Discussion:

The three dimensional structure of cellulase enzyme for Bacillus species were downloaded from PDB with accession number 4YZP for endocellulase, 5BV9 for exocellulose and 1QOX for beta-glucosidase (Figure 1 - see PDF). Similarly, polysaccharides sub units namely, cellobiose, cellotriose, cellotetraose and laminaribiose were downloaded from Pubchem database with accession number 10712, 5287993, 439626 and 439637 respectively (Figure 2 - see PDF). Using all the parameters mentioned in methodology, docking was performed for all cellulose with each polysaccharide. Each docking results in 50 conformations, from which we have selected the best docked conformations based on their binding energy, ligand efficiency, total energy and hydrogen bond formed. Figure 3 (see PDF) shows the docking complex of different cellulose component with different polysaccharide units. Considering that minimum energy reflects the more stable molecule. We have chosen the best docking results based on the binding energy, ligand efficiency, internal energy and the number of hydrogen bonds formed. Table 1 shows the results of all best docked models. Endocellulase when docked with cellobiose, cellotriose, cellotetraose and laminaribiose shows the binding energy of -7.53, -11.03, -14.21 and -11.33 respectively and ligand efficiency is almost same with all the polysaccharides. Internal energy of all docked molecules suggests that exocellulase complex with cellotetrose is most stable than other complexes with exocellulases. They are bonded by almost 4-5 hydrogen bonds in which LYS 277 and ASP 340 are mostly supporting in forming hydrogen bonds (Figure 4 - see PDF). Exocellulase when docked with cellobiose, cellotriose, cellotetraose and laminaribiose shows the binding energy of -7.33, -11.86, -14.75 and -11.22 respectively and ligand efficiency is almost same with all the polysaccharides. Internal energy of all docked molecules suggests that endocellulase complex with cellotetraose is most stable than other complexes with endocellulases. The binding of residues to polysaccharide through hydrogen bonds is not showing any significant involvement of any particular residues (Figure 5 - see PDF). Results show that exocellulase is least efficient in binding with cellobiose. Beta-glucosidase when docked with cellobiose, cellotriose, cellotetraose and laminaribiose shows the binding energy of -6.06, -12.23, -13.68 and -10.18 respectively and ligand efficiency is almost same with all the polysaccharides. Internal energy of all docked molecules suggests that endocellulase complex with cellotetraose is most stable than other complexes with endocellulases. The binding of residues to polysaccharide through hydrogen bonds is showing significant involvement of TRP225, THR297 and ASN222 (Figure 6 - see PDF). These docking result shows that cellobiose is least efficient in binding with any of the cellulase. In contrast, it is observed that cellotetraose is most efficient in binding with any of the cellulase. It is also revealed that glutamic acid, asparagine, alanine, tryptophan, tyrosine, lysine and histidine as a key residue that are involve in establishing the hydrogen bonds. This study suggests that cellulase enzyme has predominantly efficiency to bind with the cellotetraose. Our results are also supported by study conducted by Selvam *et al.* [14].

## Conclusion:

The binding affinities of exocellulase, endocellulase and beta glucosidase with cellobiose, cellotetraose, cellotetriose and laminaribiose were determined using docking scores. Results show that all components of cellulosehave highest activity against cellotetraose. We show that cellotetraose as substrate increases the conversion of cellulosic biomass for various industrial applications.

## Funding:

There are no relevant financial or non-financial competing interests to report. This research received no specific grant from any funding agency in the public, commercial or not-for-profit sectors.

## Figures and Tables

**Table 1:** Results of best docked conformations

**Cellulase**	**Polysaccharide**	**Binding energy**	**Ligand efficiency**	**Internal Energy**	**No. of H Bonds**	**H Bond Information**
Endocellulase	cellobiose	-7.33	-0.32	10.26	5	SER223, LYS200, HIS220, HIS220, TYR222
Endocellulase	cellotriose	-11.86	-0.35	6.7	4	PHE342,ASP340, LYS521, LYS277
Endocellulase	cellotetraose	-14.75	-0.33	5.45	5	LYS277,PHE342,ASP340,TRP519, PRO338
Endocellulase	laminaribiose	-11.22	-0.49	7.94	4	LYS277, LYS521, ALA339,ASP340,
Exocellulase	cellobiose	-7.53	-0.33	11.11	2	GLY20, LYS17
Exocellulase	cellotriose	-11.03	-0.32	9.51	3	TYR117,PHE176, PHE209
Exocellulase	cellotetraose	-14.21	-0.32	4.8	5	GLY179,ARG221, PRO112,GLY380, GLN180
Exocellulase	laminaribiose	-11.33	-0.49	8.07	4	THR535, ASP534, THR588, GLN533
Beta-glucosidase	cellobiose	-6.06	-0.26	9.88	3	ASN222,THR297, GLU355,
Beta-glucosidase	cellotriose	-12.23	-0.36	7.25	3	TRP225, THR297, THR223
Beta-glucosidase	cellotetraose	-13.68	-0.3	5.18	5	HIS121, TRP410, ASN356, GLU409, CYS359
Beta-glucosidase	laminaribiose	-10.18	-0.44	7.94	3	TRP225, ASN222, THR297
